# An Unusual Case of Severe Pre-eclampsia and Pulmonary Edema Following Single Fetal Demise in an In Vitro Fertilization (IVF)-Conceived Twin Gestation: A Twisted Tale of Twins

**DOI:** 10.7759/cureus.54338

**Published:** 2024-02-16

**Authors:** Smruti A Mapari, Deepti Shrivastava, Gautam N Bedi, Paschyanti R Kasat

**Affiliations:** 1 Obstetrics and Gynecology, Jawaharlal Nehru Medical College, Datta Meghe Institute of Higher Education and Research, Wardha, IND; 2 Medicine, Jawaharlal Nehru Medical College, Datta Meghe Institute of Higher Education and Research, Wardha, IND; 3 Radiodiagnosis, Jawaharlal Nehru Medical College, Datta Meghe Institute of Higher Education and Research, Wardha, IND

**Keywords:** multiple-fetus pregnancies, pre-eclampsia, pregnancy induced hypertension, twin pregnancy, single fetal death

## Abstract

There has been a notable rise in instances of multiple-fetus pregnancies over the last decade, attributed to the widespread adoption of assisted reproductive technologies. Moreover, these pregnancies have been associated with the use of drugs to induce ovulation. While some cases involve the loss of one twin with minimal consequences for the surviving twin, the demise of a fetus after the first trimester, especially beyond three months into the pregnancy, can significantly impact the health of both the mother and the surviving fetus. Unfavorable outcomes linked to the loss of one twin after the first trimester include impaired physical growth of the surviving fetus, preterm delivery, neurological abnormalities, and, in certain instances, the death of the surviving twin. This report provides a detailed account of a specific case involving twin pregnancies where a single fetal death occurred at the 24th week of gestation, leading to severe pregnancy-induced hypertension and pulmonary edema. Upon reviewing peer-reviewed articles related to similar cases in online databases, no exact matches were identified for cases with a comparable presentation. The scarcity of literature on the development of pre-eclampsia following the death of a single fetus suggests a gap in obstetric research in this area. Consequently, the uniqueness of this case report arises from its distinctive circumstances and the limited existing literature on the subject within the obstetric community.

## Introduction

It is relatively uncommon for a single fetus to experience demise within the first three months of pregnancy in twin pregnancies without significant adverse effects on the survival and development of the other fetus [1]. Nevertheless, twin pregnancies inherently pose higher risks for unfavorable outcomes affecting both maternal and fetal health. The primary contributors to perinatal morbidity and mortality in such cases are intrauterine growth retardation and premature birth [2]. A less common, yet impactful, obstetric complication associated with heightened rates of morbidity and mortality for both the fetus and the mother is the demise of a fetus following the completion of the first trimester. This occurrence has been correlated with substantial adverse effects on the physical and neurological development of the surviving fetus, resulting in additional psychological stress for both the obstetrician and the parents [3]. Furthermore, such incidents have been associated with maternal disseminated intravascular coagulation. In cases of pre-eclampsia, a condition characterized by a progressive clinical course, the only effective treatment is the delivery of the placenta [1,4]. This report presents a rare case involving dichorionic twins in a 31-year-old female who developed pre-eclampsia following the intrauterine demise of one fetus. While there are a limited number of case reports documenting the resolution of pre-eclampsia after the demise of one twin, instances of pre-eclampsia developing after the demise of a fetus have not been widely reported.

## Case presentation

**Figure 1:**
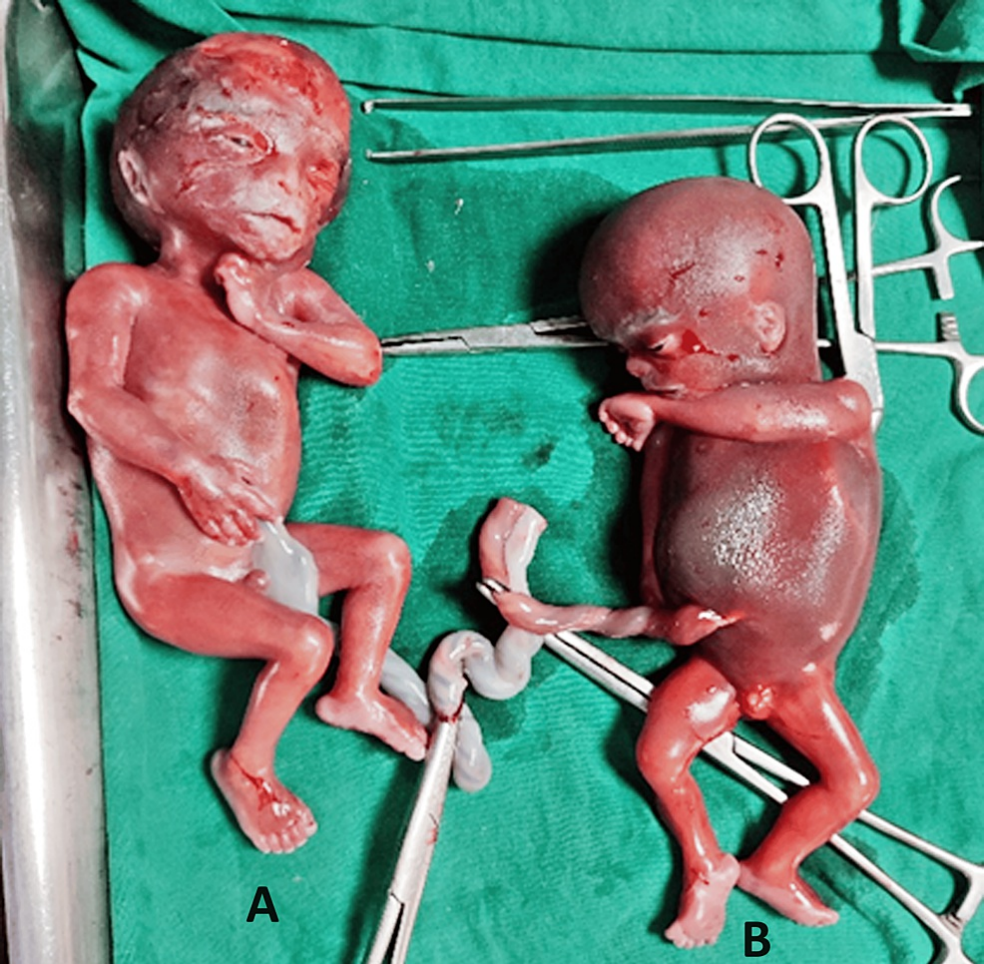
Twins delivered at 24 weeks of gestation: A) Twin Fetus; B) Fetus with IUD. IUD: Intra-uterine death.

This case presentation involves a 31-year-old G2A1 woman at 24+4 weeks of gestational age with a dichorionic diamniotic twin pregnancy conceived through in vitro fertilization. The patient, who had a cervical stitch in situ, presented for a routine antenatal check-up, during which the incidental discovery of the demise of one twin was made. The demise of the single fetus occurred at 24 weeks of gestation, resulting in severe pregnancy-induced hypertension with pulmonary edema (Figure 1).

While the patient's vital signs were stable, a per-abdomen examination revealed a detectable heart rate for only one fetus through fetal Doppler. Subsequent ultrasonography confirmed the intrauterine fetal demise of one twin and indicated intrauterine growth retardation (IUGR) in the surviving fetus. Blood investigations revealed elevated lactate dehydrogenase (LDH) and uric acid levels, while liver enzymes remained within normal limits. Additional details can be found in Table 1.

**Table 1:** Laboratory investigation of the patient.

Parameter	Patient values	Normal Range
Hemoglobin	11.2 g/DL	12-16 g/DL
Total leukocyte count	11000 /nm3	4,500-11,000 /nm3
Platelets	1.83 x 10^3^/mcL	1.5-4.5 x 10^3^ /mcL
Alanine transaminase	16 U/L	<35 U/L
Aspartate aminotransferase	37 U/L	14-36 U/L
Urea	20 mg/dl	7-17 mg/dl
Serum creatinine	0.9 mg/dl	0.52-1.04 mg/dl
Lactate dehydrogenase	496 U/L	140-280 U/L
Uric acid	6.5 mg/dl	2.5-6.5 mg/dl

The patient's medical history included hypothyroidism, for which she had been taking Tab Thyrox 37.5 mg until it was discontinued after her last delivery. She had undergone three ovum pick-ups and experienced one embryo transfer failure. Additionally, a diagnostic hysteroscopy uncovered an anterior wall septum, leading to septoplasty; a non-patent left ostia, prompting lateral wall metroplasty; and the instillation of platelet-rich plasma. The patient, admitted on the same day due to these findings, initially exhibited stable conditions and normal blood pressure. However, her blood pressure spiked to 180/110 mmHg the next day.

**Figure 2:**
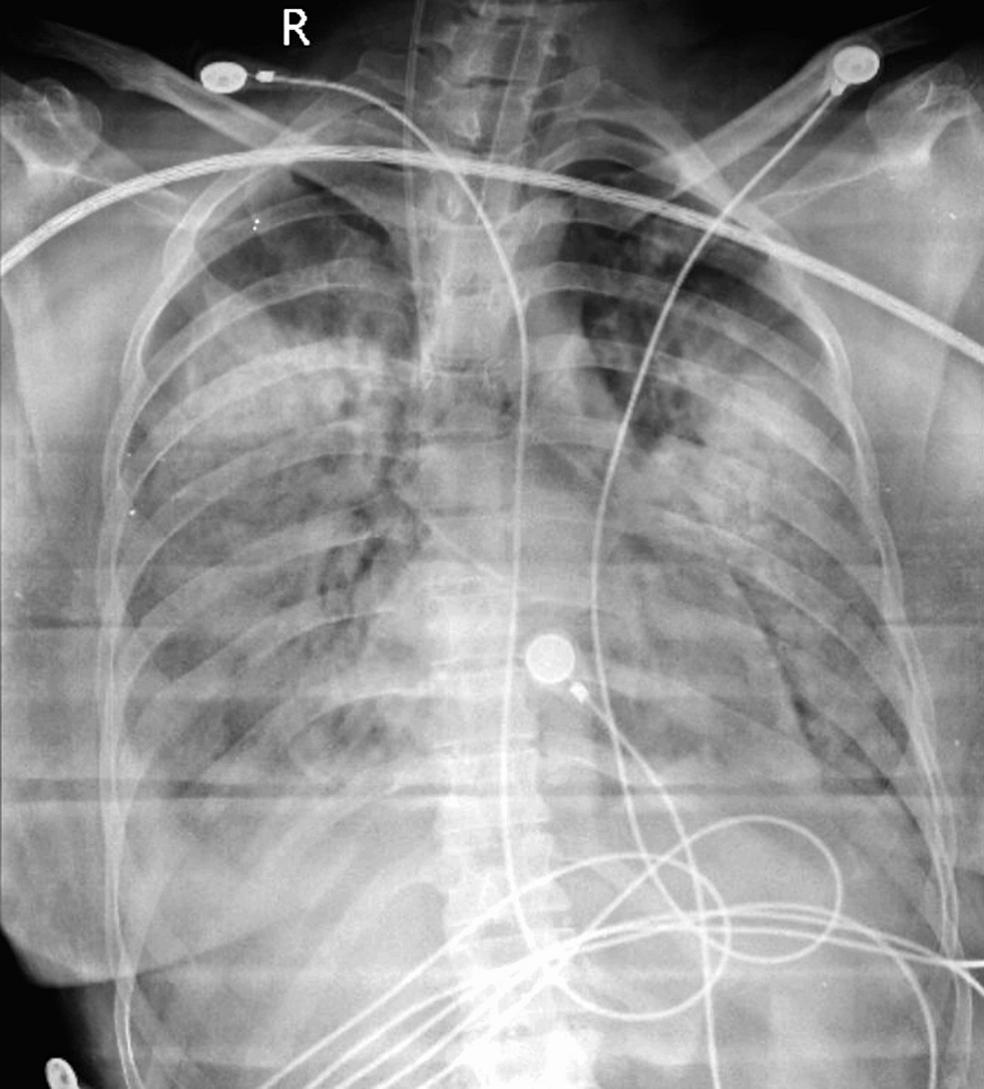
Pulmonary edema.

Upon admission, the patient was prescribed IV labetalol and prophylactic magnesium sulfate. Subsequently, she developed anasarca, prompting consultation with an emergency treating physician. The treatment plan included IV labetalol, tab Nicardia, IV Lasix, and continuous monitoring of vital signs and fetal heart rate. Complaints of breathlessness led to a chest X-ray, revealing evidence of pulmonary edema consistent with generalized anasarca (Figure 2).

**Figure 3:**
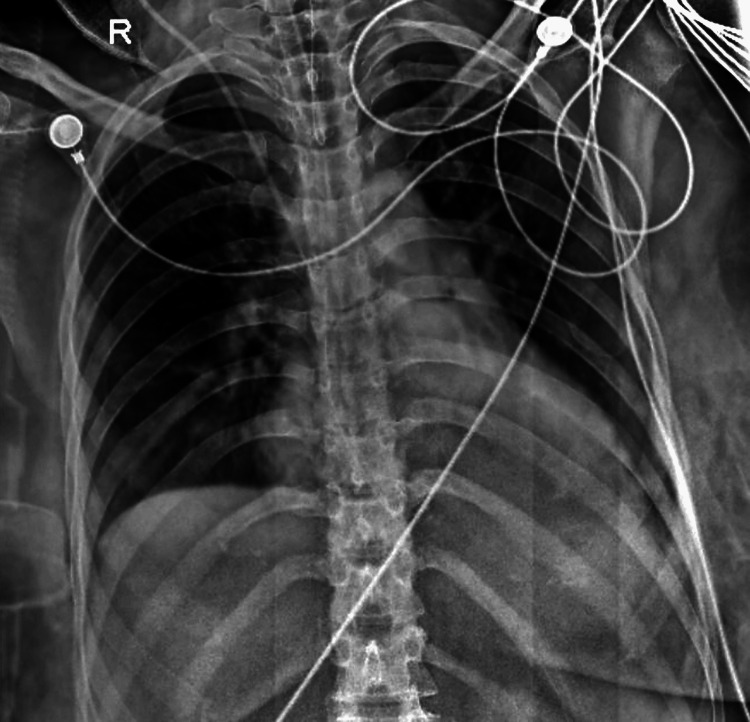
Improvement in pulmonary edema after treatment.

Despite treatment, the patient's blood pressure remained uncontrolled. Consequently, a joint decision to terminate the pregnancy for the mother's health was made. Cervical stitch removal and insertion of an intra-cervical Foley catheter with misoprostol administration were performed for cervical dilation and contractions. Twins were delivered, with one showing no signs of life post-delivery. Autopsy of both fetuses and the placenta yielded no discernible cause for fetal death. The patient's blood pressure normalized post-delivery, indicating that maternal complications, including pregnancy-induced hypertension and pulmonary edema, were associated with the demise of one fetus (Figure 3).

**Figure 4:**
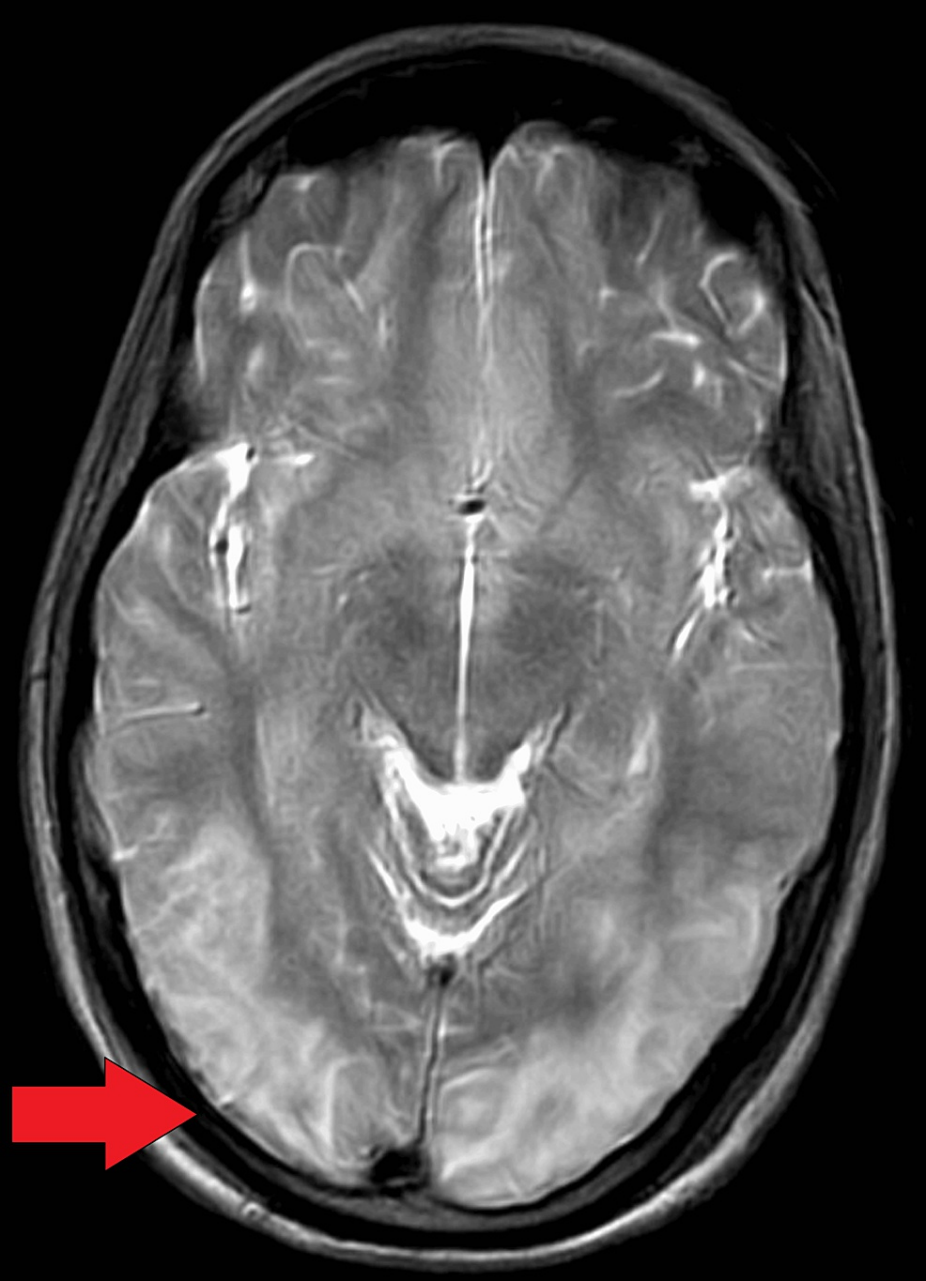
Abnormal hyperintense lesions confluent in both parieto-occipital lobes on T2-weighted MRI scan.

**Figure 5:**
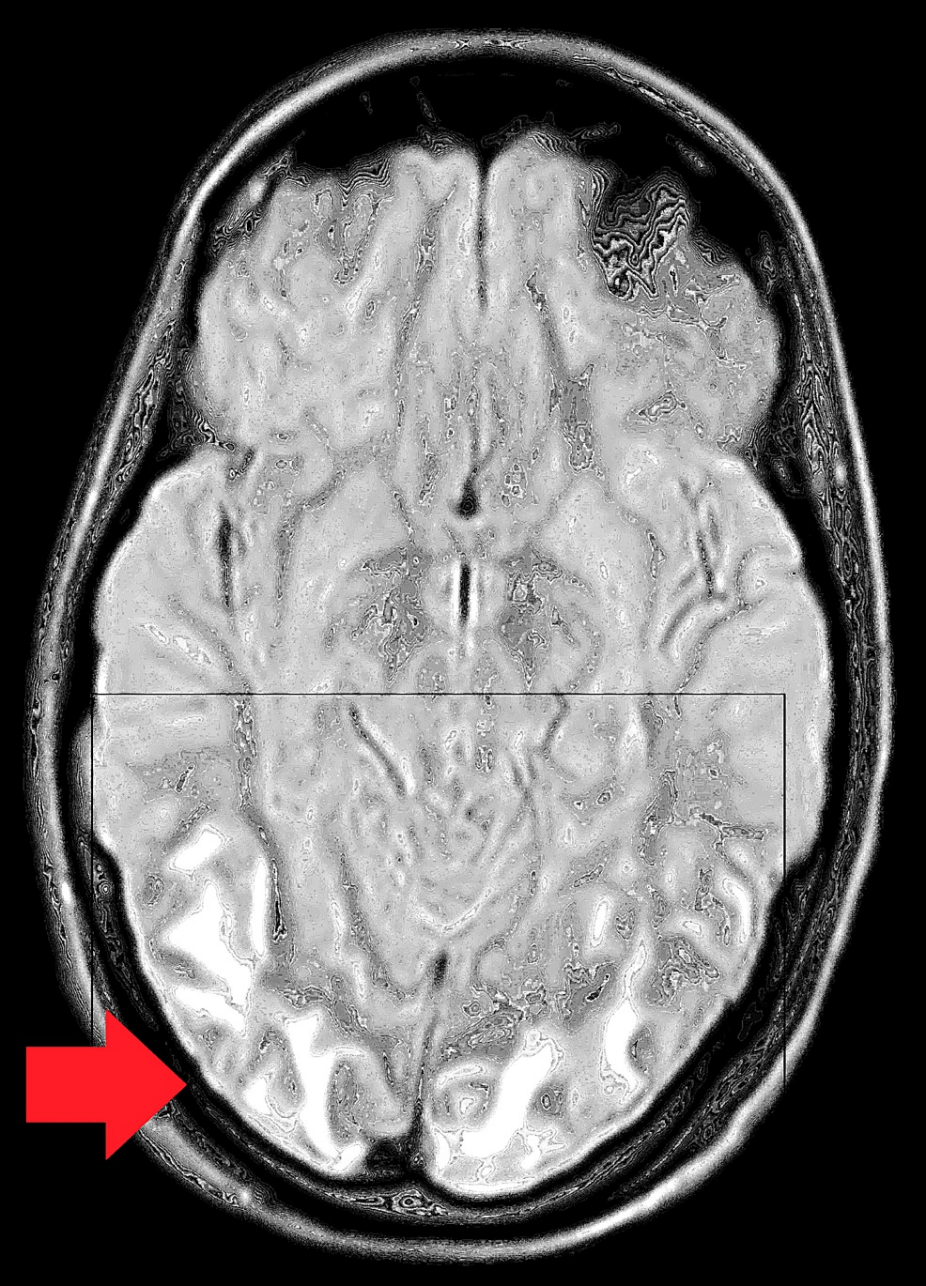
Abnormal hyperintense lesion confluent in both parieto-occipital lobes upon FLAIR scan. FLAIR: Fluid-Attenuated Inversion Recovery.

An MRI brain plain with magnetic resonance venography (MRV) revealed T2 fluid-attenuated inversion recovery (FLAIR) hyperintense foci over the bilateral corona radiata, bilateral cerebellum, left occipital cortex, midbrain, and pons. No diffusion restriction or blooming features suggestive of reversible posterior leukoencephalopathy syndrome, a common complication of hypertensive disorders of pregnancy, were observed (Figures 4-5).

## Discussion

Research studies indicate an incidence rate of 2.5% to 6% for fetal death in twin pregnancies, a figure notably higher than the 0.3% to 0.6% range observed in pregnancies with a single fetus [3-5]. In pregnancies with a single fetus, intrauterine death is reported as insignificant at any specific point during the pregnancy. In contrast, in cases of twin pregnancies, the demise of one fetus within the initial three months has relatively fewer repercussions on the development and survival of the remaining fetus. However, if fetal death occurs in the second trimester or later, significant adverse outcomes are observed for both maternal and fetal health. Such instances are associated with complications like intrauterine growth restriction (IUGR), perinatal mortality, preeclampsia, and preterm labor [5,6].

Interestingly, some cases suggest an increased survival rate for the second fetus when the death of the first fetus occurs at 33 gestational weeks [1]. Twin pregnancies, particularly those of monochorionic origin, report higher rates of single fetal deaths (50%-70%) compared to pregnancies of dichorionic origin [3]. Complications related to single fetal deaths in or after the second trimester include IUGR, chromosomal anomalies, compromised physical growth, neurological disorders, umbilical cord anomalies, and sepsis. Maternal morbidities in these cases include preeclampsia, placental abruptions, and other complications with potentially significant adverse outcomes [7-8]. Cases involving the continuation of pregnancy for more than six weeks after the death of one fetus are associated with serious complications. The most significant consequence is the potential development of diffuse intravascular coagulation (DIC), primarily caused by the release of thromboplastin and fibrin protein into the mother's bloodstream by the deceased fetus, commonly referred to as 'fetal death syndrome' [9].

The existing literature includes reports of complete resolution of preeclampsia following the death of a solitary fetus [10]. However, in our investigation, an unexpected scenario unfolded: preeclampsia manifested after the death of a single fetus. Neither of the two groups in our study experienced a case of DIC after a single intrauterine fetal death [11]. While a single intrauterine fetal death generally leads to a favorable outcome, our case deviated from this norm, as the event triggered the development of preeclampsia, further complicating the mother's health. It is crucial to counsel both parents on the necessity of a post-mortem examination of the deceased fetus, especially when the cause of death is undefined. Placental analysis for various aspects should also be considered. Given that neurological and physical abnormalities are common complications when a single fetus perishes in a twin pregnancy, a comprehensive physical and neural assessment should be conducted using various radiological and imaging modalities. This radiological screening can aid in identifying any abnormalities and managing them, with regular monitoring of postnatal fetal growth [3,7,8].

## Conclusions

This represents a rare occurrence where the development of pre-eclampsia followed the demise of a single intrauterine fetus. While typically favorable outcomes are observed after the death of a single fetus, unfortunately, our case does not align with this trend. The existing literature documents cases where pre-eclampsia completely resolves after the death of a single fetus, but our unique case, hitherto unreported, demonstrates the opposite phenomenon. It underscores the variability and complexity inherent in obstetrics, emphasizing that each pregnancy is valuable and warrants diligent efforts to achieve positive outcomes for both the mother and the fetus. This case serves as a poignant reminder that the field of obstetrics continually presents unexpected scenarios. The uniqueness of this case highlights the need for further research, particularly in the realm of twins and their intricate narratives. Recognizing the distinctiveness of this situation, there is a call for more in-depth investigations to broaden our understanding and contribute to the evolving knowledge within obstetrics.
